# A Review of Sintering-Bonding Technology Using Ag Nanoparticles for Electronic Packaging

**DOI:** 10.3390/nano11040927

**Published:** 2021-04-06

**Authors:** Jianfeng Yan

**Affiliations:** Key Laboratory for Advanced Materials Processing Technology, Ministry of Education of China, State Key Laboratory of Tribology, Department of Mechanical Engineering, Tsinghua University, Beijing 100084, China; yanjianfeng@tsinghua.edu.cn

**Keywords:** Ag NP pastes, low-temperature interconnections, sintering mechanisms, joint performances, electronic packaging

## Abstract

Metal nanoparticles (NPs) have attracted growing attention in recent years for electronic packaging applications. Ag NPs have emerged as a promising low-temperature bonding material owing to their unique characteristics. In this study, we mainly review our research progress on the interconnection of using polyol-based Ag NPs for electronic packaging. The synthesis, sintering-bonding process, bonding mechanism, and high-temperature joint properties of Ag NP pastes are investigated. The paste containing a high concentration of Ag NPs was prepared based on the polyol method and concentration. A nanoscale layer of organic components coated on the NPs prevents the coalescence of Ag NPs. The effects of organic components on the bondability of the Ag NP paste were studied. Compared to the aqueous-based Ag NP paste, the polyol-based Ag NP with the reduction of organic component can improve the bondability, and the coffee ring effect was successfully depressed due to the increased Marangoni flow. The sintering behaviors of Ag NPs during the bonding process were investigated using the classical sphere-to-sphere approach. The mechanical property of joints using this Ag paste was better than that using Pb_95_Sn_5_ solders after storage at high temperatures. The sintering–bonding technology using polyol-based Ag NPs was helpful to the low-temperature interconnection for electronic packaging applications.

## 1. Introduction

The attachment material plays an important role in the high-power electronic packaging applications, which can ensure the reliability and performance of devices [1,2,3,4,5,6]. Metal nanoparticles (NPs) have attracted growing attention in recent years owing to their unique characteristics such as low melting temperature and high diffusion coefficient, which differ from those of bulk materials [7,8,9,10,11,12]. The sintering and bonding of metal NPs at low temperatures has been demonstrated and is promising for applications in flexible electronic devices, including organic electronics and flat-panel displays [13,14,15,16].

Ag NP pastes have become the suitable choice for a new type of lead-free interconnection materials because of their high electrical conductivity, good fatigue performance, and high melting point [17,18,19,20,21]. They can work at a higher temperature than the bonding temperature with enhanced shear strength. Compared to other materials, Ag has a higher tensile strength, which can prevent the mechanical properties of Ag NP pastes from degeneration in a harsh environment. Owing to their good mechanical properties, Ag NP pastes exhibit higher bonding strength. The synthetic method and sintering process affect the bonding quality of Ag NP pastes, which dominate the application of NP pastes. It has been reported that adding organic components to Ag NPs can prevent self-aggregation in the traditional synthesis process [22,23,24,25,26]. However, this has a negative effect on sintering because the added organic components remain in the bonding layer [27]. They prevent surface and lattice diffusions from the surface and grain boundary of NPs during the sintering process, leading to a low strength bonding. There were many studies on improving the sintered properties of Ag NP pastes. To address this problem, a method for preparing Ag NP pastes based on the polyol method was proposed, and it was found that reducing the organic components in NP pastes benefitted the sintering-bonding process [8,19,27,28,29].

Therefore, this review mainly discusses improving the performance of bonding joints using the polyol-based Ag NP pastes of our research. Firstly, we introduce the progress in the synthesis of Ag NPs and the sintering-bonding process. Emphasis is placed on the polyol synthesis method and the effects of organic components on the bondability of the Ag NP paste, such as the coffee ring effect. Then, based on the sphere-to-sphere models, the sintering-bonding mechanisms were investigated. Surface diffusion and volume diffusion are found. Afterward, the applications of sintered Ag NP pastes in high-temperature environments were presented. Finally, we summarize our findings and the challenges that should be addressed for future research.

## 2. Interconnections Using Ag NP Pastes

### 2.1. Description of the Synthesis and Sintering of Ag NP Pastes

**Figure 1:**
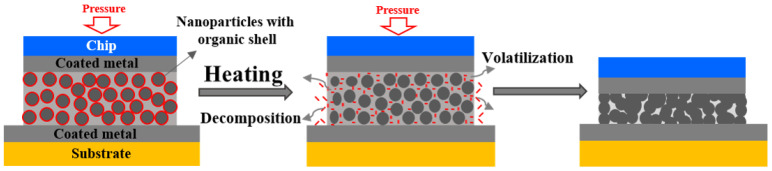
Illustration of the sintering-bonding process using polyol-based Ag NPs.

A metal NP paste is prepared by combining organic components with metal NPs to prevent agglomeration [22,23,25,26]. However, the addition of organic components inhibits the sintering process. A method for preparing high-concentration Ag NP pastes was developed based on the polyol method [30]. In the process, the NP paste was synthesized by chemical reduction, then the paste was concentrated. Figure 1 shows a schematic illustration of the bonding process using metal NP pastes in the air. First, high-concentration Ag NP pastes coated with a thin organic shell were prepared, where the organic content was lower than that of the metal-organic compound. Next, during heat processing, the organic components were decomposed and volatilized. The surface reactivity of Ag NPs re-emerged owing to the high specific surface area. Through atomic diffusion, bridges between NPs were formed, and the neck started to grow; then, the Ag NPs were sintered, promoting bonding. Note that the sintering-bonding process was dependent on the organic components, assistant pressure, and bonding temperature, which were studied in the following sections.

### 2.2. Synthesis of Ag NP Pastes and Effect of Organic Components

**Figure 2:**
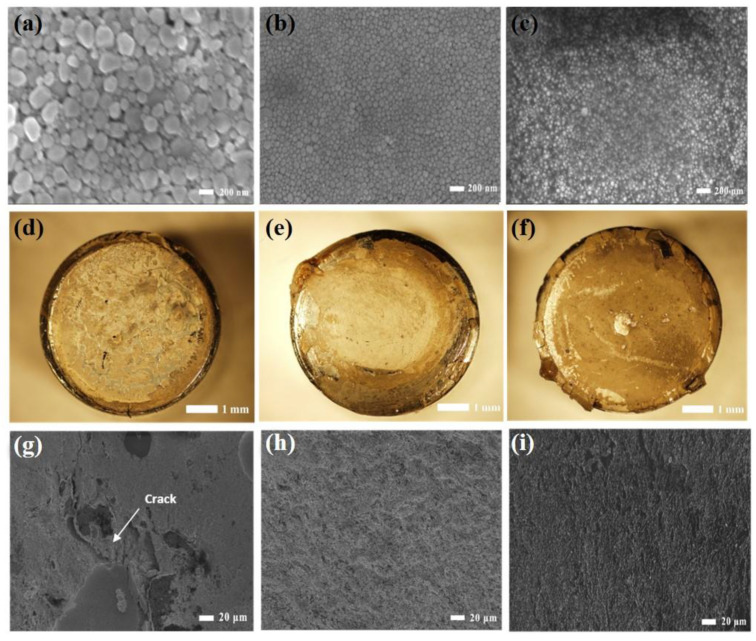
(**a**–**c**) SEM images of synthesized Ag NPs. (**d**–**f**) Optical images of the fractured bonded joints. (**g**–**i**) SEM images of the joints containing Ag NP pastes with PVP concentrations of 0.045 M, 0.45 M, 1.35 M. Reprinted/Adapted with the permission from Yan, J (2012). Copyright 2012 IOP Publishing [27].

Metal NPs have been prepared by various methods, such as chemical reduction [31], light-induced reduction [32,33], electrochemical methods [34], template methods [35], laser processing methods [36,37,38,39], and ultrasonic and microwave methods [40,41,42,43]. Table 1 summarizes the electrical conductivity of mainly typical die-attach systems, and it is easy to find that the electrical conductivities of Ag NP pastes are far higher than the other typical die-attach systems. The chemical reduction method is most commonly used in the synthesis of Ag NPs [30,44]. In this process, a solution of ethylene glycol and AgNO_3_ was prepared, along with a separate solution of ethylene glycol and polyvinyl pyrrolidone (PVP). After heating these solutions to the desired reaction temperature (160 °C), the PVP solution was dropped into the AgNO_3_ solution. Upon reaction completion, the solution was cooled by adding deionized water. Without adding organic components, the paste was prepared by concentrating the Ag NP solution. PVP was used as a protecting agent in the synthesis, which affects the properties of the resulting NP pastes and the following bonding processes [27]. Figure 2a–c shows the scanning electron microscopy (SEM) images of Ag NPs synthesized using different concentrations of PVP. With low concentrations, (0.045 M), the Ag NPs were not completely coated by PVP and tended to grow into larger NPs with diameters of 90 nm. By increasing the concentration (0.45 M), more Ag NPs were coated by PVP, and their size was reduced (diameter: 40 nm). At an even higher concentration of PVP (1.35 M), smaller NPs were obtained. Therefore, the PVP concentration clearly plays a vital role in the synthesis process of Ag NPs, preventing self- aggregation and reducing NP sizes.

**Table 1:** Electrical conductivity of mainly typical die-attach systems.

Die-Attach Systems	Electrical Conductivity, ×10^5^ (Ω cm)^−1^	Ref.
Ag NP paste	2.5	[45]
Cu micro-paste	1.3	[46]
Ag-Al NP paste	1.01	[47]
Au solder alloys	0.2–0.4	[48,49]
Sn-Pb solder alloys	0.5–0.9	[50]

The viscosities of the Ag NP solutions were measured. With the increase in PVP concentration from 0.045 M to 1.35 M, the viscosity of the Ag NP solutions increased from 4.2 cP to 6.0 cP, suggesting that the viscosity is highly dependent on the PVP concentration. In addition, the shear strengths of these Ag NP pastes were evaluated; at a bonding pressure of 5 MPa, the shear strengths were 26, 50, and 5 MPa. The concentration of PVP affects the size, viscosity, and bondability of the paste. Figure 2d–f show the fracture appearances of copper-disc joints bonded using Ag NP paste. The corresponding SEM images are shown in Figure 2g–i. For the joint using Ag NP paste with a PVP concentration of 0.045 M, the fracture is fairly rough, and many cracks are observed in the sintered layer. This results from the low viscosity of Ag NP paste. Due to cracks in the sintered layer, the average joint strength is about 26 MPa. When Ag NP pastes containing PVP concentrations of 0.45 M and 1.35 M were used as adhesive materials, dense and smooth fracture surfaces were observed. The dimple structure was easily observed, which indicates that a good joint was formed. The function of PVP in Ag NP paste is to adjust viscosity and prevent the sintered Ag layer from cracking. Because cracking of the Ag NP paste was effectively prevented, the bonding strength of the Ag NP paste with a PVP concentration of 0.45 M remarkably increased to 50 MPa, indicating that the Ag NP paste has improved adhesion with PVP incorporation. Figure 2f,i show the fracture surfaces of Ag NP pastes with a PVP concentration of 1.35 M. Given the noticeable fracture traces, the joint strength is as low as 5 MPa. According to qualitative energy-dispersive X-ray (EDX) analysis of the joint using different Ag NP pastes, only the pure Ag element was detected for Ag NP pastes with PVP concentrations of 0.045 M and 0.45 M, indicating that no organic material remained in the bonding layer. In addition to the Ag peak, a larger carbon peak appeared in the spectrum of the Ag NP paste with a PVP concentration of 1.35 M, indicating that organic matter still remains. Organic materials have a negative impact on the formation of joints because they prevent surface diffusion during the sintering, resulting in low bonding strength. The joint strength visibly decreased in this case. According to the results, PVP can prevent both the aggregation of Ag NPs and cracking of the dried Ag NP paste during the bonding process. The Ag NP paste may crack if the PVP concentration is too low. In contrast, with a high concentration of PVP, the bonding quality may be worsened by the residual organic components. An optimized PVP concentration is vital to the sintering quality of Ag NP pastes.

### 2.3. Sintering–Bonding Process of Ag NP Pastes

**Figure 3:**
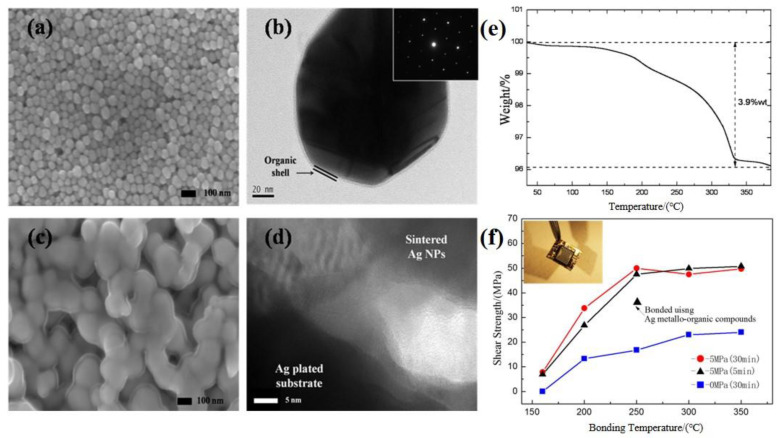
(**a**) SEM and (**b**) TEM image of Ag NPs. (**c**) SEM image of the Ag NP paste. (**d**) TEM image of the bonding interface. (**e**) Thermogravimetric analysis (TGA) of the Ag NP paste in air. (**f**) Shear strength of Ag pastes as a function of sintering temperature with external pressure. Reprinted/Adapted with the permission from Yan, J (2012). Copyright 2012 Elsevier Ltd. [8].

The sintering–bonding process of Ag NP pastes has recently become a hot topic [51]. The research objective is to develop a low-temperature bonding and enhance the bondability and robustness of joints. To prevent the aggregation of metal NPs, different organic components are added [22,23,25,26]. However, these organic components are detrimental to the sintering process. In order to remove the organic molecules, the bonding process is usually carried out at an annealing temperature of 250–350 °C with an auxiliary pressure of 1–20 MPa [19,22,23,25,26,52,53]. Nevertheless, the use of auxiliary pressure will limit the widespread application of this technology, especially in flexible electronic devices. Without auxiliary pressure, it is still possible to use highly concentrated Ag NP pastes to realize low-temperature interconnection [8]. As shown in the SEM images (Figure 3a), Ag NPs are mainly spherical with an average diameter of ≈35 nm. Figure 3b shows a transmission electron microscopy (TEM) image of Ag NPs. A transparent organic shell with a thickness of 1–2 nm can be seen on the NP surface, whose role is to prevent the aggregation of Ag NPs. Figure 3c displays the SEM image of Ag NPs (annealed at 250 °C in air for 30 min). Clearly, sintering of the NPs occurred even without external pressure, which may be caused by capillary pressure between the contacted NPs. These NPs are connected by their surfaces instead of melting completely, resulting in a denser connected structure. The sintering process is critical for the bonding. To study the interface bonding mechanism of this Ag NP paste, the bonding interface was observed by TEM at 250 °C and 5 MPa. The TEM image (Figure 3d) indicates that using the Ag NP paste as a sintering material achieves direct metal bonding.

The organic content of the Ag NP paste was characterized by thermogravimetric analysis (TGA) which is a quantitative analytical technique that monitors the mass of a sample as a furnace ramps temperature under a stable or changing gas flow, as shown in Figure 3e. When the temperature rises from 40 °C to 400 °C, the weight of the dry paste gradually decreases. The evaporation and decomposition of organic components contribute to the weight loss. According to these results, the paste is composed of ≈3.9% organic components by mass, which is much lower than the organic content found in conventional Ag paste (15% by mass) [22]. Therefore, the Ag content (96.1%) in the Ag NP paste is significantly higher than that of conventional Ag paste. It can be demonstrated that the improvement in adhesion is a result of a lower organic content because a higher content adversely affects the sintering process. The shear strength of the joints changes with the increase in bonding temperature under different pressures and holding times. As shown in Figure 3f, for holding times of 5 min and 30 min under 5 MPa, a shear strength (above 30 MPa) can be induced even at 200 °C. With the increase of bonding temperature to 250 °C, the shear strength increases to 50 MPa, demonstrating its better mechanical behavior compared to that of the conventional Ag paste [22]. Specifically, when the pressure decreases to 0 MPa, a robust joint with 10 MPa shear strength is formed at a bonding temperature of 200 °C; the shear strength increases to greater than 20 MPa at 300 °C. The variation of shear strength with bonding temperature under different pressures and holding times confirms the improved bondability of the Ag NP paste owing to the lower organic content. Additionally, an Ag NP-pasted chip bonded to a flexible circuit board is illustrated in the inset at 200 °C and 0 MPa. Thus, a pressureless bonding process can be achieved by the reduction of organic components in metal NP paste.

The bondability of robust joints is determined by uniformities of deposition layers during bonding with Ag NPs. The well-known “coffee ring effect” [54,55] influences the bonding of metal NPs at low temperatures. This effect occurs when the suspension particles are pushed to the wetting edge due to the different evaporation rates of colloid solutions. A similar phenomenon is observed for Ag NPs, in which an inhomogeneous distribution of aqueous-based pastes occurs during the bonding process at low temperatures, affecting the bonding quality. Subsequently, the mechanical strength of those joints is dramatically reduced because of the reduction in connection area. On the contrary, an improved bondability of joints has been observed when the inhomogeneous distribution of NPs is depressed during the bonding process. The introduction of polyol-based Ag paste depresses the coffee ring effect significantly because the Marangoni flow increases during the bonding process, and the mechanical strength can be improved accordingly. Ag NPs adopt different geometrical scales when synthesized via aqueous or polyol methods. The chemical reduction of AgNO_3_ is used to prepare aqueous-based Ag NPs with sodium citrate dehydrate acting as a reducing agent. The Ag NPs synthesized by the aqueous method are spherical with a mean diameter of 45 nm. The diameter of Ag NPs synthesized by the polyol method decreases to 35 nm, as shown in Table 2. Ag NP powders consist of micro-sized, flake-like pellets of tightly packed Ag NPs. These Ag NP powders can be accumulated by drying the aqueous-based Ag NP paste at 60 °C in a glass beaker.

In order to obtain a high-quality joint, it is critical to deposit the Ag NPs uniformly before joint assembly and bonding. Two kinds of Ag NP pastes (aqueous and polyol pastes) were placed on the copper substrates and evaporated in air (60 °C) to compare their deposition behavior. Coated circular areas 3–5 mm in diameter were obtained after drying the deposited Ag NPs. The aqueous Ag NPs accumulated at the rim of the coated circular areas can be attributed to the coffee ring effect [56]. After placing a droplet of Ag NP paste on the substrates, the evaporation rate at the edge was faster than that at the center [57], thus more solution evaporated from the edge. The contact line of the droplet was pinned during the evaporation, so the radius of the droplet could not shrink. The water lost at the edge could be replaced by drawing the solution from the center. The Ag NPs were carried from the center to the rim of the droplet with the water, forming a coffee ring of Ag NPs. On the other hand, the coffee ring effect could be reduced by using a polyol-based Ag NP paste. The elimination of the coffee ring can be explained by the generated Marangoni flow. The solvent for the polyol Ag NP paste is a mixture of water and ethylene glycol. Since the water and ethylene glycol have different boiling points and surface tension, Marangoni flow can be generated during the drying process of the Ag NP paste [58,59]. In addition to the convective flow, a concentration gradient was also induced by the enhanced evaporation of ethylene glycol. Owing to the different evaporation rates of water and ethylene glycol, a Marangoni-type flow from the edge to the center can also be induced. Therefore, the coffee ring effect can be reduced.

**Figure 4:**
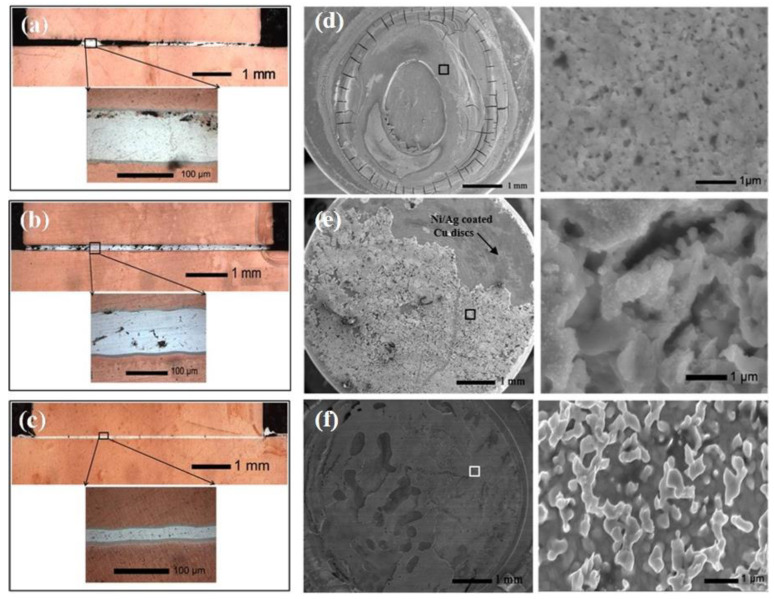
(**a**–**c**) Optical images of the joint cross-sections, and (**d**–**f**) SEM images of the shear joints using aqueous-based Ag NP paste, Ag NP powders, and polyol-based Ag NP paste. Reprinted/Adapted with the permission from Yan, J (2012). Copyright 2012 Springer Nature [61].

A uniform Ag layer is critical for good bondability. Figure 4 shows the optical images of the joints using different bonding materials. As shown in Figure 4a, a gap between Cu discs was observed in the joints using aqueous-based Ag NP pastes. The gap formation is related to the uniform deposition of the Ag NPs due to the coffee ring effect. In order to reduce this effect, dried Ag NP powders were used as the connecting material. As shown in Figure 4b, the coffee ring disappeared due to the solid state of the Ag NPs. However, voids were evident in the sintered layer of Ag NP powders. Figure 4c shows the joint using the polyol-based Ag NP paste. A dense and uniform sintered Ag layer without obvious cracks and voids was formed. This dense and uniform structure indicates that the bondability of polyol-based Ag NP paste is better than that of aqueous Ag NP paste or powder. The effects of different bonding materials on the shear strength were evaluated. Using the same bonding parameters, a drastic enhancement in the shear strength of joints is observed using the polyol-based Ag NP paste. The average shear strength using polyol-based paste reached 50 MPa, while the joints bonded using aqueous-based Ag NPs and powders possessed similar shear strengths of ≈12 MPa. Therefore, uniform deposition of the NPs plays a vital role in improving the joint strength. The distinct shear strength can be explained by the coffee ring effect and the microstructures of the shear joints. The interfacial strength is governed by the microstructures on the interface [1]. The SEM images of the microstructures on the fractured surface are shown in Figure 4d. The joints using aqueous-based Ag NPs possessed smaller connection areas due to the coffee ring effect. Although the coffee ring effect can be depressed using Ag NP powders, the shear strength remained relatively low due to the large number of voids in the sintered layer, as shown in Figure 4e. In addition, it can be seen in the SEM images that the fractured surface is partially located at the interface, which indicates a physical rather than metallurgical bonding of the sintered joints. The fractured surface of the joints using polyol-based Ag NPs is shown in Figure 4f. The coffee ring effect was prevented, and the connection area was increased by using polyol-based Ag NP paste. Denser microstructures and lower porosity at the fractured surface were observed. Both factors led to the shear strength increase. The fracture mainly occurred in the Ag NP layer, which confirms the improvement of the interfacial strength. According to the tensile failure traces, a ductile fracture mechanism was operative in the sintered NP layer [60]. Overall, the joint strength can be increased by depressing the inhomogeneous distribution of NPs.

### 2.4. Sintering Mechanisms of Ag NP Pastes

**Figure 5:**
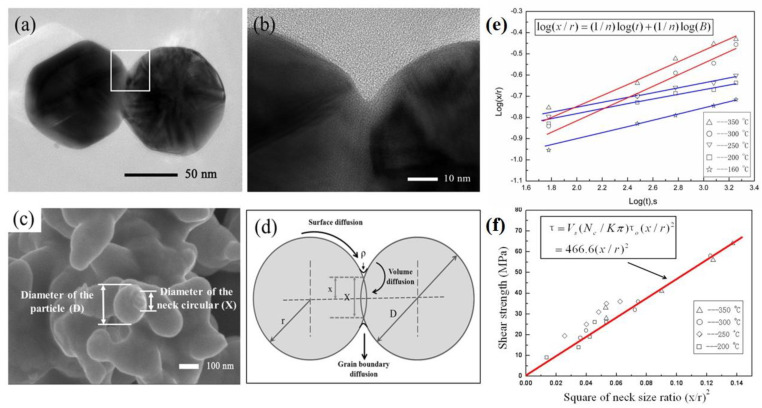
(**a**,**b**) TEM images and (**c**) SEM image of the sintering of two adjacent Ag NPs. (**d**) Schematic of the sphere model, where D is the diameter of the NP and X is the diameter of the interparticle neck. (**e**) Neck growth kinetics at different temperatures. (**f**) The relationship between the joint strength and the square of the neck size ratio (x/r)^2^. Reprinted/Adapted with the permission from Yan, J (2015). Copyright 2015 Springer Nature [71].

An understanding of the underlying mechanisms of metal NP sintering is necessary in the fields of electric packaging and manufacturing [8,22,53,62,63,64,65,66,67,68,69,70]. The sintering process of Ag NPs has been discussed using the classical sphere-sphere model [71], and the relationship between the mechanical properties and microstructures was investigated. The sintering process of NPs is crucial in the formation of bonding joints because fractures typically occur in the sintered NP layer [22,53]. The sample was prepared by sintering Ag NPs in air. As shown in Figure 5a–c, two spherical Ag NPs were sintered, and the “neck” between the two was formed after heating.

Multiple models have been developed in order to explain the bonding of NPs [72,73,74], among which the classical sphere model proposed by Frenkel focused on the contact of two metal NPs [72]. As shown in Figure 5d, at the initial stage, the model consists of two spheres having equal sizes. The neck between the two spheres (with a diameter of D and radius *r*) is assumed to be a circle (with a diameter of *X* and radius *x*). According to the sintering theory, the neck growth is explained by different material transport processes. Theoretical equations for different sintering mechanisms (viscous flow, volume diffusion, grain boundary diffusion, and surface diffusion) have been reported [72], [74,75]. The sintering equations for different sintering mechanisms can be generally expressed as follows [74]:

(1) $${\left(x/r\right)}^{n}=Bt$$

where *x*/*r* is the ratio of the interparticle neck radius to the particle radius; *B* is a constant related to geometry, particle size, sintering temperature, and nature of the material; *t* is the sintering time; and *n* is a mechanism-dependent exponent determined by the material transport process (viscous flow: *n* = 2; volume diffusion: *n* = 4–5; grain boundary diffusion: *n* = 6; surface diffusion: *n* = 7).

The exponent *n* is determined by the sintering mechanism. Therefore, by measuring *n*, the dominant sintering mechanisms of neck growth can be studied. According to Equation (1), a logarithmic formulation of the sintering equation can be derived:

(2) log(x/r)=(1/n)log(t)+(1/n)log(B)

Since *B* is a constant, the inverse slope of the linear fitting between the log(*x*/*r*) and log(*t*) values is the exponent *n*. Figure 5e shows the logarithmic evolution curves of the interparticle neck size ratio (*x*/*r*) with sintering time at different sintering temperatures. The solid line is the linear fitting of log(*x*/*r*) and log(*t*). Two sintering mechanisms are found. At a sintering temperature of 160, 200, and 250 °C, the values of the inverse slope are 6.7, 8.8, and 8.4, respectively. Consequently, surface diffusion could be the dominant sintering mechanism at temperatures between 160 °C and 250 °C. The high specific surface activity of Ag NPs at low sintering temperature contributes the surface diffusion. At higher sintering temperature, volume diffusion becomes the dominant diffusion mechanism. The inverse slope of the line at 300 °C and 350 °C is 3.7 and 3.8, respectively, which coincide with the characteristic exponent of volume diffusion.

The organics in the Ag NP paste also affect the sintering mechanisms, according to the Fourier-transform (FTIR) spectrum of the Ag NP paste after sintering at different temperatures. The peaks at 2953 cm^−1^ and 1677 cm^−1^ correspond to the C–H stretch and C=O stretch, respectively. After sintering at 160 °C, the characteristic peak of C=O is red-shifted to 1633 cm^−1^. The red shift of the C=O peak indicates that the carboxyl oxygen atom of PVP interacts with the Ag NP surface [76]. At sintering temperatures of 300 and 350 °C, the peak at 2893 cm^−1^ (C–H stretch) disappears, and the peak at 1032 cm^−1^ (C–N stretch) is significantly weakened. These results indicate that the PVP is decomposed at this sintering temperature. When the temperature is below 250 °C, the PVP was still coated on the Ag NP, and surface diffusion was the dominant sintering mechanism. When the temperature increases to above 300 °C, the PVP in the Ag NP paste is destroyed, and the sintering process changes to volume diffusion. Consequently, the decomposition of organics in the Ag NP paste may affect the sintering mechanism. By adjusting the amount and type of organic components in paste, the sintering behaviors and bondability of Ag NPs can be controlled. Because the alkylamine will evaporate at 130 °C, using alkylamine as a dispersant will facilitate the sintering process of Ag NPs.

As a key property of the joint, it is important to study the joint strength of the Ag NP paste after sintering. Empirical and theoretical analyses indicate that the microstructures of sintered materials affect the mechanical properties of joints [77,78]. Fracturing of joints mainly occurs in the sintered layer. Therefore, the microstructure of the sintered Ag NP paste is related to the joint strength. An obvious fracture is found at the interparticle bonds. The joint strength of Ag NP paste is mainly determined by the sintered bond between contacting Ag NPs [79]. The sphere-sphere model of particle sintering has been used to analyze the sintering mechanism of NPs as well as the relationship between the strength and microstructure. The joint strength depends on the intrinsic material strength and the ratio of the sintered neck area to the cross-section area through the spherical center. In order to determine the relationship between the joint strength of the Ag NP paste and the ratio of the sintered neck area to the cross-section area through the spherical center, a sintering joint experiment was carried out at a temperature of 250–300 °C, and the relationship between the micromorphology of the Ag NP joint and the joint strength was analyzed (Figure 5f). It can be seen that there is a linear relationship between the strength of the Ag NP joint and (*x*/*r*)^2^, which represents the size of the sintering neck. According to the sintering model, it can be concluded that the degree of joint bonding between adjacent particles after sintering is one of the main sources of strength. It can be inferred that a higher interface connection ratio leads to higher joint strength [80].

### 2.5. High-Temperature Joint Properties of Ag NP Pastes

The metal NPs have a high specific surface activity, and thus show a low sintering temperature. This type of high-performance bonding material is suitable for both high- and low-temperature bonding environments and has broad application prospects. At present, the bonding material is often used for devices in high-temperature environments and broadband gap semiconductor devices [62,65,66,67,81]. Therefore, it is interesting to study the high-temperature properties of joints utilizing polyol-based Ag NP paste. For this reason, the joint bonded with Ag NP paste was stored at temperatures of 200–350 °C for 50 h before evaluating the joint properties [82]. The results indicate that the high-temperature properties of the joint bonded with Ag NP paste are superior to those of a joint in which Pb_95_Sn_5_ was applied.

**Figure 6:**
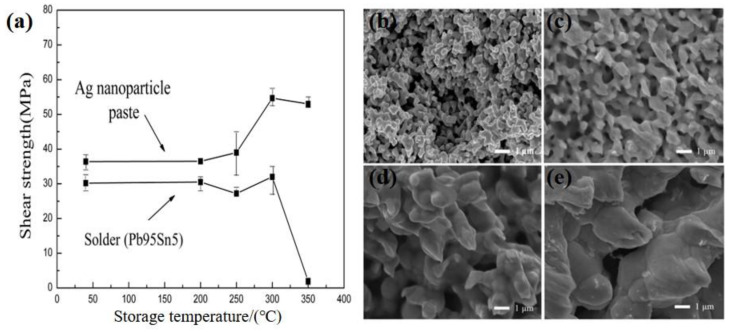
(**a**) Comparison of the joint strength using Ag NP paste and Pb_95_Sn_5_ solder after storage at different temperatures for 50 h. SEM fracture surfaces of the joints using Ag NP paste at different temperatures of (**b**) 200, (**c**) 250, (**d**) 300 and (**e**) 350 °C. Reprinted/Adapted with the permission from Yan, J (2016). Copyright 2016 Hindawi Publishing [82].

The influence of temperature, which ranges from 200–350 °C, on the shear strength of the joints bonded with Pb_95_Sn_5_ solder and Ag NP paste are shown in Figure 6a. The strength of the joints using Ag NP paste rises after heat treatment at 250, 300, and 350 °C, which can be attributed to the further sintering. The neck growth and grain growth observed in the fracture microstructures can also explain the strength increase of the joint bonded with Ag NP paste (Figure 6b–e). The strength of the joint bonded with Pb_95_Sn_5_ solder does not noticeably change until the temperature rises to 350 °C, when the strength decreases to 0 and the joint disconnects. Through EDX analysis, Sn, Ag, Cu, and O were detected on the surface of the disconnected joint, which implies the breakage of the Ni/Ag layer plated on the Cu substrate and the occurrence of oxidation. This is due to the fact that 350 °C is higher than the melting point. Paknejad et al. summarized the shear strengths of joints using Ag NP paste after heat treatment and found that the strength increases at a treatment temperature below 300 °C in most situations [83]. However, if the temperature is too high, oxygen may penetrate into the sintered Ag due to its porous structure. For example, the Cu substrate without a plated layer can oxidize within 24 h at 300 °C [84]. The collective results suggest that the joint bonded with Ag NP paste has good high-temperature properties, which are likely superior to those of the joint coated with Pb_95_Sn_5_ solder. In addition, the Ag NP paste has some other advantages compared with the other commonly used interconnection materials. Table 3 summarizes the property comparison of some commonly used solder alloys with the Ag NP paste.

## 3. Summary and Future Trends

This review summarized our research progress on the sintering–bonding technology using Ag NPs for electronic packaging applications. A low-temperature bonding method using polyol-based Ag NPs was developed. The deposition behavior of the NP layer related to the coffee ring effect was studied. Strong joints were formed using Ag pastes. The reduction of organic components in the paste was helpful for achieving low-temperature bonding. The classical sphere-sphere model was introduced to investigate the sintering mechanisms of Ag NPs during the sintering process. Different sintering mechanisms, which could be related to the decomposition of organic components, were revealed at different sintering temperatures. The joints bonded with Ag NP pastes showed good resistance to high temperature, indicating that the Ag NP paste could be used in high-temperature environments. This interconnection technology using metal NP pastes has shown potential application for electronic packaging. More researches should be carried out to further promote the applications in the future:

(1) Generally, in order to obtain robust joints with a high shear strength, the sintering-bonding process is usually carried out with an auxiliary pressure, which limits widespread applications, especially in flexible electronic devices. Future work needs to focus on reducing the pressure applied on chips during the sintering processes.
(2) The bonding between NP pastes and metal substrates is a complicated process, which relies on multiple factors, such as organic components, sintering temperature, pressure, and paste deposition distribution. Future work needs to focus on the interfacial reactions between NP pastes and metal base during the sintering processes.
(3) The classical sphere-to-sphere models are usually used to investigate the sintering-bonding mechanism of NP pastes. For other pastes which have various morphologies, the sintering-bonding mechanism could be different. Future work needs to focus on the study of the theoretical models.

## Figures and Tables

**Table 2:** Properties of Ag NP pastes prepared with three different methods.

	Size Diameter	Coffee Ring Effect	Joints	Shear Strength (MPa)
Aqueous-based Ag NPs	45 nm	Existence	Gaps	12
Polyol-based Ag NPs	35 nm	Elimination	No defects	50
Ag NP powders	Micro-sized	Elimination	Voids	12

**Table 3:** Property comparison of some commonly used solder alloys with Ag NP pastes. Reprinted/Adapted with the permission from Zhang, P (2018). Copyright 2018 Elsevier Ltd. [70].

	Pb_37_Sn_63_	Sn_96.5_Ag_3.5_	Au_80_Sn_20_	Ag NP Pastes
Bonding mechanism	Liquidus reflow	Liquidus reflow	Liquidus reflow	Sintering
Maximum use temperature (°C)	180	220	280	960
Electrical conductivity ×10^5^ (Ω cm)^−1^	0.69	0.82	0.62	2.6
Thermal conductivity (W/mK)	51	60	58	240
Elastic modulus (GPa)	16	26	68	10
Yield strength (MPa)	27	22.5	N/A	43
Tensile strength (MPa)	27	52	275	43

## Data Availability

The data presented in this study are available in article.
